# Ab Initio Calculation of the Zn Isotope Effect in Phosphates, Citrates, and Malates and Applications to Plants and Soil

**DOI:** 10.1371/journal.pone.0030726

**Published:** 2012-02-17

**Authors:** Toshiyuki Fujii, Francis Albarède

**Affiliations:** 1 Research Reactor Institute, Kyoto University, Osaka, Japan; 2 Ecole Normale Supérieure, Lyon, France; KULeuven, Belgium

## Abstract

Stable Zn isotopes are fractionated in roots and leaves of plants. Analyses demonstrate that the heavy Zn isotopes are enriched in the root system of plants with respect to shoots and leaves as well as the host soil, but the fractionation mechanisms remain unclear. Here we show that the origin of this isotope fractionation is due to a chemical isotope effect upon complexation by Zn malates and citrates in the aerial parts and by phosphates in the roots. We calculated the Zn isotope effect in aqueous citrates, malates, and phosphates by *ab initio* methods. For pH<5, the Zn isotopic compositions of the various parts of the plants are expected to be similar to those of groundwater. In the neutral to alkaline region, the calculations correctly predict that ^66^Zn is enriched over ^64^Zn in roots, which concentrate phosphates, with respect to leaves, which concentrate malates and citrates, by about one permil. It is proposed that Zn isotope fractionation represents a useful tracer of Zn availability and mobility in soils.

## Introduction

Using XAFS microspectroscopy [1], it was found that Zn is dominantly bound to phosphate in the root system of the pseudo-metallophyte *Arabidopsis halleri* and to malate and citrate in the aerial parts. The importance of this observation was strengthened by the remarkable discovery [2] that ^66^Zn in the root system of plants grown in a controlled environment is enriched over ^64^Zn by 0.6 permil (‰) with respect to the aerial parts of the plant, an observation replicated and confirmed on plants collected in their natural habitat [3]–[5]. Because zinc isotopic variability in the natural environment is relatively narrow, with the exception of FeMn-hydroxide deposits, Zn isotope variability in plants is reflected in soils [5], [6]. In addition, it is well established that the absorption and desorption of both Zn and phosphates are strongly responsive to the pH of groundwater, hence the implications for water pollution are particularly important [7]. Zinc isotopes therefore have the potential to be a tracer of soil status and evolution as well as of water quality. Isotope fractionation by hydrated Zn ion, chlorides, sulfides, sulfates, carbonates, and citrates has recently been evaluated by *ab initio* methods [8], [9]. Here we set out to calculate by the same techniques Zn isotope fractionation for phosphates and malates and extend previous results for citrates. We then discuss the implications for plant physiology and soil status.

## Materials and Methods

### Theoretical background

Isotopic exchange in chemical reactions can be represented by two half-reactions,
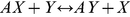
(1)or
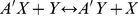
(2)where A and A′ are the heavy and light isotopes of the element A, and X and Y represent ligands. The difference between half-reactions 1 and 2 corresponds to a reaction of isotopic exchange between AX and AY:
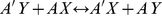
(3)The isotope separation factor α between AX and AY is defined as
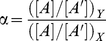
(4)where ([A]/[A′])_X_ and ([A]/[A′])_Y_ are the isotopic ratios A/A′ measured in the complexes AX (and A′X) and AY (and A′Y), respectively. The isotope enrichment factor is defined as α_m_−1. Since α is close to 1, α−1 can be approximated as ln α.

Deviations of isotopic ratios from a reference value in parts per 1000 are conventionally defined as
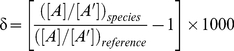
(5)If AX (and A′X) is the major component in the system, Σ[A]/Σ[A′] is approximated to be ([A]/[A′])_X_ such that an approximation expression δ≈10^3^ ln α is suitable.

The standard theory of chemical isotope fractionation is based on mass-dependent isotopic differences in vibrational energies of isotopologues [10], [11]. The isotope enrichment factor is proportional to 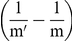 with *m* and *m*′ the masses of two isotopes (prime represents the light isotope).

The isotope enrichment ln α due to intramolecular vibrations can be evaluated from the reduced partition function ratio (RPFR) β = (*s*/*s*′)*f* defined as
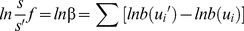
(6)where the sum extends over all the molecular vibrational level with primed variables referring to the light isotopologue and
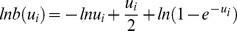
(7)In this equation, ν*_i_* stands for vibrational frequencies, *s* for the symmetry number of the molecule, and *u_i_* = *h*ν*_i_*/*kT*. The isotope enrichment factor due to the molecular vibration can be evaluated from the frequencies summed over all the different modes. The partition function ratio (*s/s*′)*f* for isotopologues A′X and AX (A′Y and AY, respectively) is noted β_X_ (β_Y_, respectively). In the isotopic exchange reaction 3, isotope fractionation can be estimated from the relation ln α≈ln β_Y_−ln β_X_. Contribution of other isotope effects, such as the nuclear field shift effect, to ln β is less than 10% for the Zn chloride system [12]. An adequate approximation of fractionation factors between different Zn species may be obtained by the conventional mass-dependent theory. All the calculations were made for the ^66^Zn/^64^Zn ratio.

In the present study, the optimized structures of Zn species were first determined for ^64^Zn. The intramolecular vibrational frequencies ν_i_ were calculated for each complex. ln *b*(*u_i_*′) was determined by substituting ν_i_ into Eq. (7). Then ^64^Zn was replaced by ^66^Zn and the vibrational frequencies were calculated again for the same molecular structures to obtain ln *b*(*u_i_*), from which ln β was then determined.

### Computational details

Orbital geometries and vibrational frequencies of aqueous Zn(II) species were computed using density functional theory (DFT) as implemented by the Gaussian09 code [13], [14]. The DFT method employed here is a hybrid density functional consisting of Becke's three-parameter non-local hybrid exchange potential (B3) [15] with Lee-Yang and Parr (LYP) [16] non-local functionals. In a quantum chemical study, the convergence of the reaction energies of Zn(II) species is excellent in 6–311+G(d,p) or higher basis sets [17]. Hence, the 6–311+G(d,p) basis set, which is an all-electron basis set, was chosen for H, C, O, P, and Zn. The geometry optimization and intramolecular vibrational frequency analysis were performed for the hydrated Zn ion, hydrated Zn citrates, hydrated Zn malates, and hydrated Zn phosphates. For hydrated Zn ion, the results were reproduced from our previous studie [9], [12]. Molecules were modeled without any forced symmetry. An “ultrafine” numerical integration grid was used and the SCF convergence criterion was set to 10^−9^.

## Results

**Figure 1:**
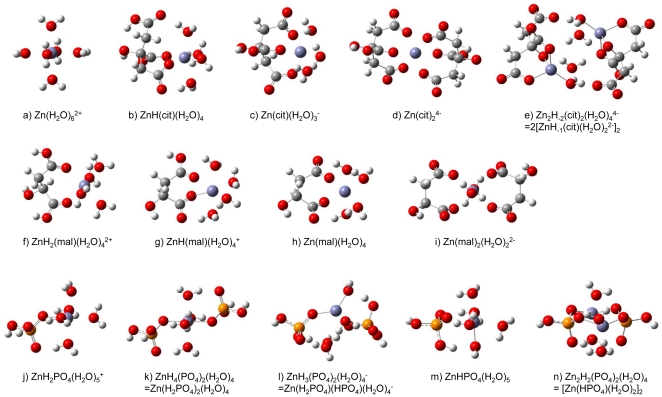
Molecular structures of hydrated Zn^2+^ and aqueous Zn citrates, malates, and phosphates. Structures are drawn by using GaussView5 (Gaussian Inc.) [14]. Symbols are: Zn (iris), P (orange), O (red), C (gray), and H (white).

The possible Zn species are shown in **Figure 1** and the ln β values obtained are shown in **Table 1**. The optimized structure Cartesian coordinates are given in the **File S1**. The hydrated Zn^2+^ ion possesses six H_2_O molecules in its first coordination shell and complexation with large anions such as Cl^−^ decreases the coordination number from six to four (see [12]). In the present study, we therefore assumed that the coordination number of Zn species is six. Coordination with multidentate ligands, however, sometimes decreases the coordination number due to stereochemical restriction.

**Table 1:** Logarithm of the reduced partition function, ln β (‰), for the isotope pair ^66^Zn/^64^Zn.

Species	Temperature (K)					
	273	298	323	373	473	573
Zn(H_2_O)_6_^2+^	3.854	3.263	2.797	2.119	1.334	0.915
ZnH(cit)(H_2_O)_4_	4.033	3.419	2.934	2.227	1.406	0.967
Zn(cit)(H_2_O)_3_^−^	4.154	3.523	3.024	2.297	1.452	0.999
Zn(cit)(H_2_O)_3_^−^ a)	−	3.94a)	−	−	−	−
Zn(cit)_2_^4−^	2.889	2.437	2.083	1.572	0.986	0.675
Zn_2_H_−2_(cit)_2_(H_2_O)_4_^4−^	5.330	4.523	3.884	2.953	1.867	1.284
ZnH_2_(mal)(H_2_O)_4_^2+^	3.842	3.250	2.784	2.107	1.325	0.909
ZnH(mal)(H_2_O)_4_^+^	3.984	3.376	2.896	2.197	1.386	0.952
Zn(mal)(H_2_O)_4_	4.103	3.479	2.987	2.268	1.433	0.986
Zn(mal)_2_(H_2_O)_2_^2−^	3.274	2.771	2.376	1.801	1.135	0.780
ZnH_2_PO_4_(H_2_O)_5_^+^	4.092	3.468	2.975	2.257	1.424	0.978
ZnH_4_(PO_4_)_2_(H_2_O)_4_	4.047	3.428	2.940	2.229	1.405	0.965
ZnH_3_(PO_4_)_2_(H_2_O)_4_^−^	5.027	4.268	3.667	2.789	1.764	1.214
ZnHPO_4_(H_2_O)_5_	4.188	3.559	3.060	2.330	1.476	1.017
Zn_2_H_2_(PO_4_)_2_(H_2_O)_4_	5.156	4.380	3.765	2.865	1.814	1.249

a) Initial input configuration was taken from a model molecule “ZnCit01” of the literature [8]. The LanL2DZ basis set was chosen for Zn and the 6–31G(d) basis set for H, C, and O. ln β = 3.93‰ at 298 K was reported.

Four citrate species, Zn(cit)^−^, ZnH(cit)^0^, Zn(cit)_2_^4−^, and Zn_2_H_−2_(cit)_2_^4−^, were found in a titration measurement [18]. The molecular structure and ln β for hydrated Zn monocitrate, Zn(cit)(H_2_O)*_n_* ^−^, have been estimated by the *ab initio* method [8]. Our calculations, which reproduced these results, were expanded to Zn(cit)_2_^4−^, hydrated ZnH(cit), and hydrated Zn_2_H_−2_(cit)_2_^4−^. Since citrate ion (cit)^3−^ is a tridentate ligand, Zn(cit)(H_2_O)_3_^−^ and Zn(cit)_2_(H_2_O)_0_^4−^ were calculated as hydrated Zn(cit)^−^ and Zn(cit)_2_^4−^, respectively. The monoprotonated citrate ion H(cit)^2−^ showed a bidentate character, such that four H_2_O were arranged in the empty coordination sites of ZnH(cit). The deprotonated citrate ion H_−1_(cit)^4−^ is a strong anionic ligand. Calculations show that the Zn^2+^coordination number for the dimeric species Zn_2_H_−2_(cit)_2_^4−^ must be reduced to four. Zn_2_H_−2_(cit)_2_(H_2_O)_4_^4−^, in which two H_2_O bind to Zn^2+^ and another two bridge the H_−1_(cit)^4−^ ligands, was optimized.

Several species of Zn malates were found in titration studies [19], [20]. According to these authors, ZnH_2_(mal)^2+^, ZnH(mal)^+^, and Zn(mal)^0^ emerge as the potential malate species. A higher-order complexation of Zn(mal)_2_^2−^ may exist [20]. Malate ion (mal)^2−^ and its protonated species were treated as bidentate ligands, with H_2_O molecules arranged in the empty coordination sites of Zn malates.

Complexation of Zn in orthophosphate solutions has been studied in a pioneering work using titration method [21]. The results were reexamined and several different species were found [22]. Literature data suggest the existence of five Zn phosphate species, ZnH_2_PO_4_^+^, ZnH_4_(PO_4_)_2_, ZnH_3_(PO_4_)_2_^−^, ZnHPO_4_, and Zn_2_H_2_(PO_4_)_2_. Except for strong acidic/basic conditions (pH<3 and pH>11), the hydrogen phosphate ions, H_2_PO_4_^−^ and HPO_4_^2−^, are the major anionic species in orthophosphate solutions. Cadmium ion, Cd^2+^, a congener ion of Zn^2+^, in orthophosphate solutions shows a sixfold coordination, in which five coordination sites are occupied by H_2_O and one by an oxygen atom of the hydrogen phosphate ion [23]. Hence, H_2_PO_4_^−^ and HPO_4_^2−^ are treated as monodentate ligands. Since the bonding power of HPO_4_^2−^ is stronger than that of H_2_PO_4_^−^, HPO_4_^2−^ attracts H^+^ from a H_2_O molecule in ZnHPO_4_(H_2_O)_5_ and the optimized structure resembles that of ZnH_2_PO_4_(H_2_O)_4_OH. For ZnH_4_(PO_4_)_2_, *cis*- and *trans*- configurations of Zn(H_2_PO_4_)_2_(H_2_O)_4_ were tested and the *trans*-configuration was energetically stable. Calculations of ZnH_3_(PO_4_)_2_^−^ as Zn(H_2_PO_4_)(HPO_4_)(H_2_O)_4_ deformed six coordination to smaller values. Dehydrated H_2_O molecules bridged H_2_PO_4_^−^ and HPO_4_^2−^. Configuration of dimeric species such as Zn_2_H_2_(PO_4_)_2_ has been suggested in the literature [21] and was applied in this study.

## Discussion

**Figure 2:**
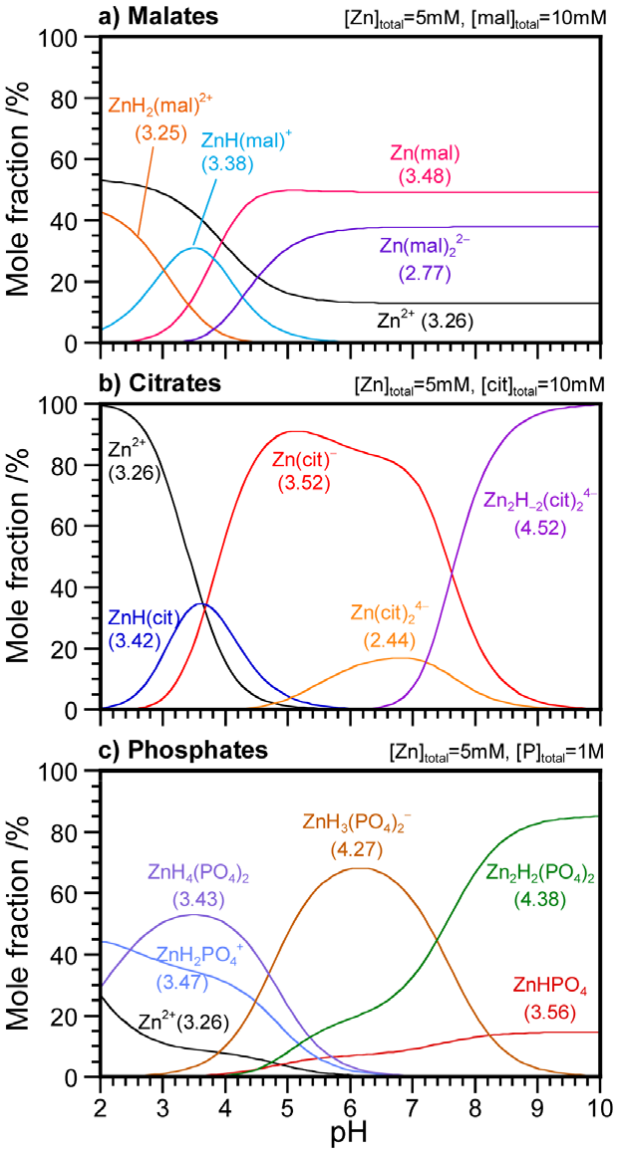
Mole fractions of Zn species as functions of pH at 298 K. Mole fractions of Zn species were calculated by using formation constants and acid dissociation constants reported in the literature [18], [20]–[22]. The ln β values at 298 K are shown in parentheses. a) Mole fractions of Zn species in malate solutions. Total concentrations of Zn and malate are 5 mM and 10 mM, respectively. b) Mole fractions of Zn species in citrate solutions. Total concentrations of Zn and citrate are 5 mM and 10 mM, respectively. c) Mole fractions of Zn species in orthophosphate solutions. Total concentrations of Zn and phosphate are 5 mM and 1 M, respectively.

**Figure 3:**
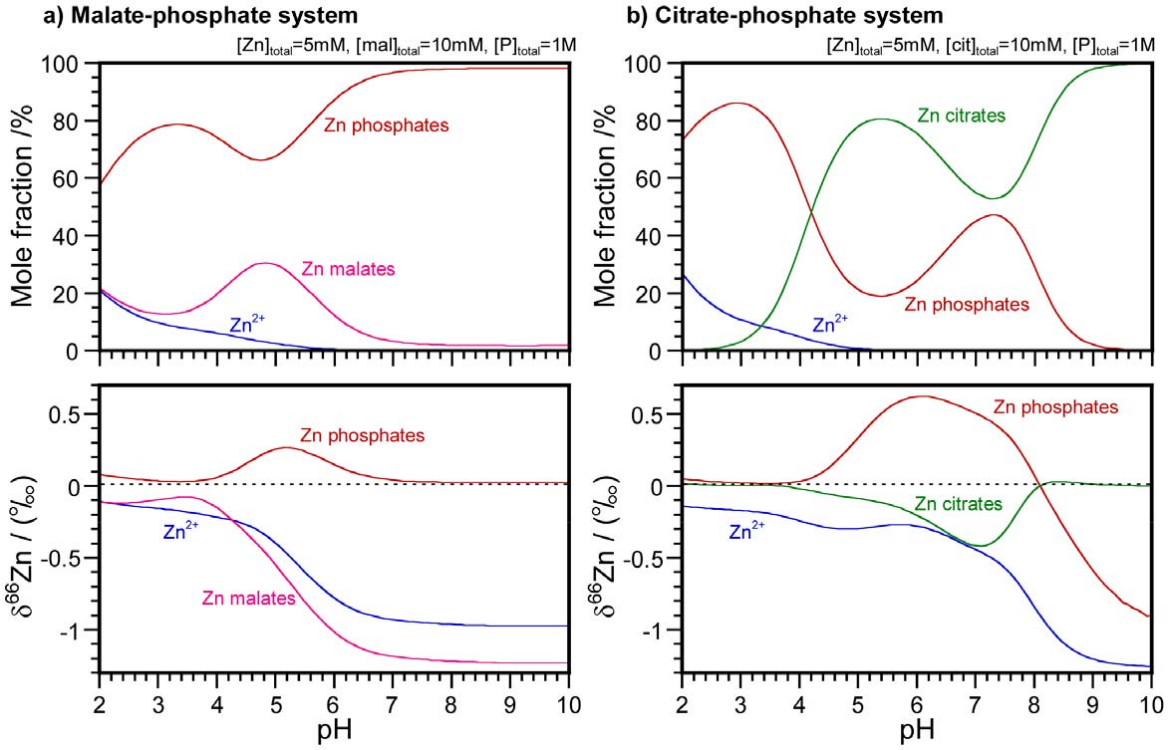
Isotope fractionation δ^66^Zn of hydrated Zn^2+^, and aqueous citrates, malates, and phosphates at 298 K. Mole fractions of Zn species were calculated by using formation constants and acid dissociation constants reported in the literature [18], [20]–[22]. Isotope fractionation δ^66^Zn (‰) compared with the bulk solution (averaged δ^66^Zn in the whole solution, δ^66^Zn = 0) is shown together as a dotted line. The calculation procedure of δ^66^Zn is from [9]. a) Malate-phosphate system. Total concentrations of Zn, malate, and phosphate are 5 mM, 10 mM, and 1 M, respectively. b) Citrate-phosphate system. Total concentrations of Zn, citrate, and phosphate are 5 mM, 10 mM, and 1 M, respectively.

Mole fractions of Zn citrates, malates, and phosphates are shown in **Figure 2** as functions of pH. These values were calculated from literature data on formation constants [18], [20]–[22] and acid dissociation constants [18], [20], [21]. As an example, concentrations of Zn and citrate were set to the values in the literature [18], i.e., [Zn]_total_ = 0.005 mol dm^−1^ (M) and [cit] _total_ = 0.01 M. Similar conditions were chosen for malates. For the phosphate system, we kept [Zn]_total_ at the same level as in the other systems and used a rather high phosphate concentration of [P]_total_ = 1 M. In **Figure 2**, the ln β values at 298 K (see **Table 1**) are shown in parentheses. Isotope fractionation δ^66^Zn (‰) is the difference between the ln β values of the different species. We calculated δ^66^Zn between Zn^2+^, Zn citrates, malates, and phosphates (**Figure 3**). Let us first discuss ^66^Zn/^64^Zn fractionation between these species, at acidic, neutral, and basic regions. In the low pH region (pH<5), ln β values of all species are tightly grouped between 3.26 and 3.47‰. In the neutral region, ln β is 4.3–4.4‰ for the major phosphate species and ∼3.5‰ for Zn citrates and malates. The difference is therefore about 0.8–0.9‰. Fractionation with respect to the original solution with positive δ^66^Zn in phosphates and negative δ^66^Zn in citrates and malates therefore is expected. In the basic region (pH>8), the major phosphate and citrate species show ln β∼4.4–4.5‰, while Zn malates and Zn^2+^ show ln β<3.5‰. The difference is about 0.9–1‰. Negative δ^66^Zn in malates and positive δ^66^Zn in phosphates and citrates are again expected. Overall, phosphates preferentially concentrate isotopically heavy Zn, whereas Zn^2+^ and malates concentrate the light Zn isotopes. Isotope fractionation in citrates varies from positive to negative depending on pH but does not seem to alter either the phosphate/malate or the phosphate/Zn^2+^ systems. Zinc isotope fractionation between phosphate and Zn^2+^ and between phosphate and malate therefore is of the order of 1‰ at pH>5. The presence of citrates may affect both Zn speciation and δ^66^Zn, but for pH<8, phosphate isotopically remains the heaviest Zn compound.

Using EXAFS and x-ray microfluorescence, it was pointed out that for a number of *Arabidopsis* species, Zn is mostly distributed among phosphates, malates and citrates, with phosphates being present notably in the root system [1]. In situ determination of pH by pseudoradiometric methods demonstrated that, cytoplasmic pH is generally stable at around 7.2 [24]. Both spectrometric and isotopic evidence [2], [3], [5] indicate that Zn phosphates must be the species responsible for Zn isotope fractionation between the root system, rich in phosphates and characterized by isotopically heavy Zn, and the aerial parts, rich in malate and characterized by isotopically light Zn. Phosphate may also account for the high δ^66^Zn of herbaceous plants with respect to nutrient solutions [5]. The presence of phosphate with a preferential uptake of heavy Zn explains why leachable Zn in soils has high δ^66^Zn [25], whereas residual silicates have low δ^66^Zn [5], [6].

Nevertheless, pH at the spot of root hair initiation can drop below 4.5 [26], [27]. Low pH are consistent with the finding [4] that the root system of the Zn hyperaccumulator *Arabidopsis halleri* has much higher δ^66^Zn than the root system of the nonaccumulator *Arabidopsis petraea*. A simple explanation of this observation is that the pH is regulated around a neutral value by the root system of *Arabidopsis halleri*, which promotes phosphate dissociation and Zn complexation. In contrast, in the root system of *Arabidopsis petraea*, pH drops to values pH<5 Zn, with the consequence that phosphate complexation is minimal and Zn isotope fractionation is greatly reduced.

In general, the fates of Zn and phosphate seem to be strongly connected and Zn isotopes should provide a new perspective on the chemistry of soils and groundwater. Phosphates are adsorbed on iron hydroxide precipitating in seawater [28], estuaries [29], and, at pH<7, soils [30]. Adsorption of Zn-phosphate liberated by the drainage of soils appears to be a straightforward explanation for the high δ^66^Zn of ferromanganese nodules [31]. Potential applications to the origin of field experiments [7] involving injection of solutions with well-controlled chemistry and pH into a polluted soil show that Zn and P are released coherently when the pH of the solution falls below 5, which is consistent with the release of Zn initially bound in phosphate. Natural Zn leached from plant roots should be isotopically heavy, whereas the composition of a pollutant should reflect Zn from ores and be substantially lighter.

### Conclusions

We investigated fractionation of ^66^Zn and ^64^Zn between phosphates, malates, and citrates, three Zn compounds abundant in plants, using *ab initio* techniques for a broad range of pH conditions. We found that, for pH>5, Zn phosphate is 1‰ heavier than Zn^2+^ and Zn malate and, for pH<8, the heavy character of Zn phosphates is not greatly affected by the presence of citrates. This result accounts for the high δ^66^Zn found for roots and herbaceous plants with respect to leaves and sprouts.

## Supporting Information

File S1 Optimized structure Cartesian coordinates of hydrated Zn^2+^ ion, citrates, malates, and phosphates (see Figure 1).
(DOC)
