# Supplementary material for: Ab Initio Calculation of the Zn Isotope Effect in Phosphates, Citrates, and Malates and Applications to Plants and Soil
Source: PLoS One. 2012 Feb 17;7(2):e30726. doi: 10.1371/journal.pone.0030726 (PMC3281869; doi:10.1371/journal.pone.0030726)
Supplement: File S1 — Optimized structure Cartesian coordinates of hydrated Zn2+ ion, citrates, malates, and phosphates (see Figure 1). (DOC) [file pone.0030726.s001.doc]

**Supporting Information**

# Ab Initio Calculation of the Zn Isotope Effect in Phosphates, Citrates, and Malates and Applications to Plants and Soil

# Toshiyuki Fujii^1^* and Francis Albarède^2^

*^1^ Research Reactor Institute, Kyoto University, 2-1010 Asashiro Nishi, Kumatori, Sennan, Osaka 590-0494, Japan*

*^2^ Ecole Normale Supérieure de Lyon, Université de Lyon 1, CNRS, 46, Allee d'Italie, 69364 Lyon Cedex 7, France*

Table S1. Optimized structure Cartesian coordinates of hydrated Zn^2+^ ion, citrates, malates, and phosphates (see Figure 1).

Zn(H_2_O)_6_^2+^

| Element | X | Y | Z |
| --- | --- | --- | --- |
| Zn | 0.000026 | 0.000014 | -0.000010 |
| O | 0.612463 | -1.838912 | -0.878282 |
| O | -1.384498 | -1.050191 | 1.228861 |
| O | 1.503404 | -0.217831 | 1.489653 |
| O | 1.384546 | 1.050202 | -1.228858 |
| O | -1.503503 | 0.217749 | -1.489545 |
| O | -0.612487 | 1.838946 | 0.878196 |
| H | 2.303939 | 1.251699 | -1.006328 |
| H | 1.212923 | 1.409191 | -2.110385 |
| H | 0.270221 | -2.717425 | -0.662548 |
| H | 1.280310 | -1.948331 | -1.569258 |
| H | -1.212869 | -1.409192 | 2.110381 |
| H | -2.303892 | -1.251681 | 1.006332 |
| H | -1.682200 | -0.393479 | -2.217494 |
| H | -2.125345 | 0.954279 | -1.568664 |
| H | -1.280341 | 1.948337 | 1.569170 |
| H | -0.270256 | 2.717469 | 0.662488 |
| H | 2.125240 | -0.954366 | 1.568780 |
| H | 1.682084 | 0.393386 | 2.217617 |

ZnH(cit)(H_2_O)_4_

| Element | X | Y | Z |
| --- | --- | --- | --- |
| Zn | 1.705062 | 0.189012 | 0.340915 |
| O | -0.054250 | -0.415080 | 1.140503 |
| O | -2.198057 | -0.419264 | 1.777509 |
| O | 0.816999 | 1.492700 | -0.996706 |
| O | 0.013001 | 3.026719 | 0.416948 |
| O | -2.346051 | -2.720885 | -1.128971 |
| O | -0.155618 | -2.878499 | -1.413510 |
| O | -3.198817 | -0.155157 | -0.557094 |
| O | 2.092716 | -1.573860 | -0.780525 |
| O | 2.313772 | -0.991136 | 2.105720 |
| O | 2.122416 | 1.939070 | 1.554445 |
| O | 3.472481 | 0.975644 | -0.801300 |
| C | -1.795536 | 0.135745 | -0.503201 |
| C | -1.573804 | 1.657270 | -0.742845 |
| C | -1.144598 | -0.691068 | -1.630100 |
| C | -1.317069 | -0.266536 | 0.938755 |
| C | -1.157495 | -2.182194 | -1.380069 |
| C | -0.155958 | 2.128817 | -0.420278 |
| H | -2.274655 | 2.210600 | -0.118362 |
| H | -1.803537 | 1.873449 | -1.791035 |
| H | -0.129898 | -0.355786 | -1.811492 |
| H | -1.726166 | -0.495781 | -2.538399 |
| H | 2.700043 | -1.474128 | -1.521366 |
| H | 1.310852 | -2.113243 | -1.082148 |
| H | 1.376394 | -1.158891 | 2.322363 |
| H | 2.688342 | -1.844261 | 1.855973 |
| H | 2.008959 | 1.839757 | 2.506102 |
| H | 1.412013 | 2.567035 | 1.230695 |
| H | -3.464960 | -0.186884 | 0.385050 |
| H | -2.996428 | -1.991611 | -1.019751 |
| H | 4.005485 | 1.516614 | -0.205226 |
| H | 2.863012 | 1.599098 | -1.237638 |

Zn(cit)(H_2_O)_3_^−^

| Element | X | Y | Z |
| --- | --- | --- | --- |
| Zn | 1.248829 | -0.095737 | 0.137457 |
| O | -0.345977 | 0.068609 | 1.406419 |
| O | -2.504919 | -0.044318 | 1.970248 |
| O | 0.657209 | 1.258843 | -1.316235 |
| O | 0.185909 | 3.052827 | -0.074981 |
| O | -1.244938 | -3.116365 | -0.608522 |
| O | 0.594878 | -1.848033 | -0.669080 |
| O | -3.462887 | 0.209916 | -0.381546 |
| O | 2.834953 | -0.469340 | -1.451762 |
| O | 1.792376 | -1.639705 | 1.745975 |
| O | 2.139342 | 1.611100 | 1.147547 |
| C | -2.038637 | 0.258536 | -0.359566 |
| C | -1.652616 | 1.700492 | -0.833681 |
| C | -1.509024 | -0.844138 | -1.355433 |
| C | -1.603944 | 0.065576 | 1.137176 |
| C | -0.698595 | -2.042818 | -0.820868 |
| C | -0.173151 | 2.064987 | -0.740421 |
| H | -2.227435 | 2.413122 | -0.242023 |
| H | -1.977713 | 1.784349 | -1.876354 |
| H | -0.901482 | -0.356938 | -2.118188 |
| H | -2.406819 | -1.251886 | -1.817509 |
| H | 2.480559 | 0.285354 | -1.947223 |
| H | 2.280020 | -1.222588 | -1.718659 |
| H | 0.987670 | -1.384530 | 2.227528 |
| H | 1.462572 | -2.291672 | 1.097437 |
| H | 1.777313 | 1.579134 | 2.041341 |
| H | 1.614294 | 2.327536 | 0.697913 |
| H | -3.700138 | 0.078949 | 0.556970 |

Zn(cit)(H_2_O)_3_^−^ ^a)^

| Element | X | Y | Z |
| --- | --- | --- | --- |
| Zn | 1.335927 | -0.090840 | 0.142195 |
| O | -0.313121 | 0.052426 | 1.387526 |
| O | -2.481387 | 0.053135 | 1.964613 |
| O | 0.691859 | 1.262375 | -1.316967 |
| O | 0.187303 | 3.004439 | 0.002486 |
| O | -1.224785 | -2.964860 | -0.197793 |
| O | 0.659251 | -1.869232 | -0.742643 |
| O | -3.435264 | 0.195575 | -0.392270 |
| O | 2.800703 | -0.440743 | -1.524725 |
| O | 1.768042 | -1.709869 | 1.637419 |
| O | 2.078897 | 1.609212 | 1.267387 |
| C | -2.017132 | 0.256237 | -0.376467 |
| C | -1.627920 | 1.692449 | -0.876931 |
| C | -1.464699 | -0.859418 | -1.344168 |
| C | -1.576821 | 0.094071 | 1.122351 |
| C | -0.656271 | -2.005403 | -0.706597 |
| C | -0.151162 | 2.049242 | -0.728753 |
| H | -2.225682 | 2.412866 | -0.313321 |
| H | -1.922155 | 1.751363 | -1.932572 |
| H | -0.856625 | -0.392200 | -2.120578 |
| H | -2.356980 | -1.298967 | -1.795550 |
| H | 2.410023 | 0.338291 | -1.968423 |
| H | 2.179418 | -1.170100 | -1.749228 |
| H | 0.947373 | -1.458752 | 2.110138 |
| H | 1.418495 | -2.290443 | 0.913168 |
| H | 1.514017 | 1.490956 | 2.052554 |
| H | 1.575199 | 2.315765 | 0.754046 |
| H | -3.648854 | 0.123685 | 0.567061 |

^a)^ Initial input configuration was taken from a model molecule "ZnCit01" of the literature [1]. The LanL2DZ basis set was chosen for Zn and the 6-31G(d) basis set for H, C, and O.

Zn(cit)_2_^4−^

| Element | X | Y | Z |
| --- | --- | --- | --- |
| Zn | 0.000000 | 0.000000 | 0.000000 |
| O | 1.828668 | -0.055844 | -1.157157 |
| O | 3.959128 | -0.192175 | -1.834761 |
| O | 0.693496 | -1.705311 | 1.114394 |
| O | 1.761089 | -3.236753 | 2.355887 |
| O | 2.317602 | 2.206900 | 2.979715 |
| O | 0.995337 | 1.240524 | 1.448709 |
| O | 5.035171 | -0.558487 | 0.372474 |
| O | -1.828668 | 0.055844 | 1.157157 |
| O | -3.959128 | 0.192175 | 1.834761 |
| O | -0.693496 | 1.705311 | -1.114394 |
| O | -1.761089 | 3.236753 | -2.355887 |
| O | -2.317602 | -2.206900 | -2.979715 |
| O | -0.995337 | -1.240524 | -1.448709 |
| O | -5.035171 | 0.558487 | -0.372474 |
| C | 3.579848 | -0.426461 | 0.519800 |
| C | 3.156450 | -1.780297 | 1.143369 |
| C | 3.421510 | 0.808564 | 1.442113 |
| C | 3.045525 | -0.202787 | -0.946445 |
| C | 2.105777 | 1.441579 | 1.997093 |
| C | 1.728727 | -2.243801 | 1.575378 |
| C | -3.579848 | 0.426461 | -0.519800 |
| C | -3.156450 | 1.780297 | -1.143369 |
| C | -3.421510 | -0.808564 | -1.442113 |
| C | -3.045525 | 0.202787 | 0.946445 |
| C | -2.105777 | -1.441579 | -1.997093 |
| C | -1.728727 | 2.243801 | -1.575378 |
| H | 3.491979 | -2.552977 | 0.439204 |
| H | 3.787624 | -1.906632 | 2.029716 |
| H | 4.042127 | 0.601723 | 2.320762 |
| H | 3.923812 | 1.636024 | 0.923998 |
| H | 5.101367 | -0.451182 | -0.607331 |
| H | -3.491979 | 2.552977 | -0.439204 |
| H | -3.787624 | 1.906632 | -2.029716 |
| H | -4.042127 | -0.601723 | -2.320762 |
| H | -3.923812 | -1.636024 | -0.923998 |
| H | -5.101367 | 0.451182 | 0.607331 |

Zn_2_H_−2_(cit)_2_(H_2_O)_4_^4−^

| Element | X | Y | Z |
| --- | --- | --- | --- |
| Zn | -0.343902 | 0.791997 | 2.353593 |
| Zn | 0.343939 | -0.792050 | -2.351980 |
| O | -2.615120 | 1.755705 | 5.601564 |
| O | -0.955427 | 1.154308 | 4.240321 |
| O | 0.411797 | 2.744568 | -0.162753 |
| O | 1.516650 | 1.181305 | 1.788892 |
| O | -1.715538 | 1.800351 | 1.419168 |
| O | -3.654611 | -1.198850 | 1.933801 |
| O | -1.439030 | -0.853636 | 1.947538 |
| O | -3.333874 | 0.533673 | -1.143637 |
| O | -5.135480 | 1.867346 | -1.121095 |
| O | 2.613153 | -1.760234 | -5.600358 |
| O | 0.954334 | -1.157300 | -4.238686 |
| O | -0.412270 | -2.745943 | 0.162246 |
| O | -1.517265 | -1.179618 | -1.788386 |
| O | 1.716029 | -1.799245 | -1.417373 |
| O | 3.655413 | 1.198751 | -1.938514 |
| O | 1.439715 | 0.854112 | -1.949395 |
| O | 3.335523 | -0.530026 | 1.143263 |
| O | 5.137547 | -1.863131 | 1.121042 |
| C | -2.182335 | 1.470803 | 4.467062 |
| C | -3.219219 | 1.551187 | 3.309481 |
| C | -2.870945 | 1.122724 | 1.831896 |
| C | -2.679034 | -0.448256 | 1.879491 |
| C | -4.098385 | 1.509317 | 0.974234 |
| C | -4.170483 | 1.278461 | -0.563993 |
| C | 2.181071 | -1.474129 | -4.465894 |
| C | 3.218680 | -1.553256 | -3.308832 |
| C | 2.871318 | -1.122442 | -1.831755 |
| C | 2.679661 | 0.448536 | -1.881920 |
| C | 4.099066 | -1.507929 | -0.974050 |
| C | 4.171932 | -1.275212 | 0.563923 |
| H | -3.499976 | 2.610998 | 3.264212 |
| H | -4.103109 | 1.007403 | 3.658897 |
| H | -4.260264 | 2.584018 | 1.116222 |
| H | -4.981118 | 1.003516 | 1.380879 |
| H | 2.181518 | 0.482450 | 1.486572 |
| H | 1.357109 | 1.801538 | 1.034922 |
| H | -0.487899 | 2.453459 | 0.101708 |
| H | 0.693453 | 2.179679 | -0.913388 |
| H | 3.498999 | -2.613129 | -3.262167 |
| H | 4.102571 | -1.010342 | -3.659577 |
| H | 4.260827 | -2.582822 | -1.114716 |
| H | 4.981683 | -1.002722 | -1.381689 |
| H | -2.181543 | -0.480143 | -1.486060 |
| H | -1.358887 | -1.800541 | -1.034957 |
| H | 0.487433 | -2.454915 | -0.102213 |
| H | -0.693704 | -2.180335 | 0.912459 |

ZnH_2_(mal)(H_2_O)_4_^2+^

| Element | X | Y | Z |
| --- | --- | --- | --- |
| Zn | 2.134527 | 0.997969 | -2.031782 |
| O | -0.098541 | -0.743116 | 1.075417 |
| O | 0.982588 | 0.482614 | -0.354800 |
| O | 2.022780 | 3.052130 | -1.441763 |
| O | 2.392538 | -1.023975 | -2.659131 |
| O | 3.768554 | 0.583122 | -0.722373 |
| O | 3.071234 | 1.795871 | -3.788880 |
| O | -1.535545 | 1.193339 | -4.155236 |
| O | 0.422394 | 1.024728 | -3.193078 |
| O | -2.891446 | 1.499252 | -2.056986 |
| C | -0.085329 | -0.027475 | -0.027813 |
| C | -1.376690 | 0.099031 | -0.808818 |
| C | -1.522479 | 1.331877 | -1.741390 |
| C | -0.795950 | 1.169411 | -3.089956 |
| H | -1.517579 | -0.829761 | -1.375131 |
| H | -2.215043 | 0.146426 | -0.108084 |
| H | 4.693607 | 0.862150 | -0.724154 |
| H | 3.542093 | 0.323160 | 0.181748 |
| H | 3.091294 | -1.604762 | -2.331391 |
| H | 2.048967 | -1.406103 | -3.477006 |
| H | 2.523735 | 1.811804 | -4.586865 |
| H | 3.993277 | 1.765652 | -4.077195 |
| H | 2.089734 | 3.380793 | -0.535744 |
| H | 2.361704 | 3.743387 | -2.026384 |
| H | -1.101831 | 2.221080 | -1.260760 |
| H | -3.304553 | 2.217131 | -1.561619 |
| H | -0.969095 | -1.098562 | 1.312307 |
| H | -2.473202 | 1.333126 | -3.897225 |

ZnH(mal)(H_2_O)_4_^+^

| Element | X | Y | Z |
| --- | --- | --- | --- |
| Zn | 2.184113 | 0.922874 | -2.107065 |
| O | 0.098611 | 1.585357 | 0.856880 |
| O | 0.967999 | 0.001970 | -0.390551 |
| O | 2.674359 | 2.358850 | -0.512795 |
| O | 1.943950 | -0.548746 | -3.575341 |
| O | 4.004382 | 0.124316 | -1.395722 |
| O | 3.106441 | 2.360933 | -3.423673 |
| O | -0.596748 | 0.028473 | -3.652098 |
| O | 0.387296 | 1.693165 | -2.497158 |
| O | -2.959468 | 0.472287 | -2.383404 |
| C | -0.048179 | 0.560992 | -0.008849 |
| C | -1.422936 | 0.233329 | -0.513183 |
| C | -1.795697 | 1.015874 | -1.846115 |
| C | -0.593045 | 0.909959 | -2.782682 |
| H | -1.448133 | -0.830743 | -0.748147 |
| H | -2.200493 | 0.445681 | 0.223482 |
| H | 4.260659 | 0.543147 | -0.562710 |
| H | 4.237932 | -0.809974 | -1.340057 |
| H | 2.161462 | -1.486876 | -3.549600 |
| H | 0.980017 | -0.459028 | -3.838224 |
| H | 2.442950 | 2.741189 | -4.015767 |
| H | 3.879506 | 2.149818 | -3.962283 |
| H | 1.996245 | 2.390212 | 0.180624 |
| H | 2.851201 | 3.267614 | -0.786812 |
| H | -1.976776 | 2.065466 | -1.603869 |
| H | -2.698672 | -0.144540 | -3.084905 |
| H | -0.750471 | 1.956076 | 1.136345 |

Zn(mal)(H_2_O)_4_

| Element | X | Y | Z |
| --- | --- | --- | --- |
| Zn | 2.185027 | 0.999210 | -2.028631 |
| O | 0.387599 | 1.539960 | 0.651706 |
| O | 1.117180 | -0.108130 | -0.676842 |
| O | 2.588138 | 2.396888 | -0.443107 |
| O | 2.065610 | -0.376291 | -3.713440 |
| O | 3.821249 | -0.150476 | -1.123785 |
| O | 3.023329 | 2.336912 | -3.546784 |
| O | -0.544767 | 0.093989 | -3.741517 |
| O | 0.425063 | 1.833637 | -2.708092 |
| O | -2.851783 | 0.475274 | -2.377945 |
| C | 0.174042 | 0.585900 | -0.117120 |
| C | -1.239249 | 0.244856 | -0.554625 |
| C | -1.664584 | 1.027769 | -1.848811 |
| C | -0.527592 | 0.987280 | -2.873345 |
| H | -1.300045 | -0.819552 | -0.785976 |
| H | -1.955509 | 0.490972 | 0.229470 |
| H | 4.367554 | 0.456800 | -0.608503 |
| H | 3.193442 | -0.534453 | -0.485461 |
| H | 2.250856 | -1.304128 | -3.531715 |
| H | 1.089433 | -0.310208 | -3.928766 |
| H | 2.138917 | 2.628775 | -3.827099 |
| H | 3.349726 | 1.787263 | -4.271108 |
| H | 1.808281 | 2.169130 | 0.161173 |
| H | 2.525676 | 3.337139 | -0.642723 |
| H | -1.860851 | 2.067785 | -1.583044 |
| H | -2.573238 | -0.145625 | -3.067588 |

Zn(mal)_2_(H_2_O)_2_^2−^

| Element | X | Y | Z |
| --- | --- | --- | --- |
| Zn | 0.000000 | -0.000027 | -0.000018 |
| O | -2.217702 | -0.195537 | 3.181159 |
| O | -1.181859 | -1.079606 | 1.383841 |
| O | -0.479562 | -1.945096 | -1.098322 |
| O | -3.026032 | -0.823693 | -1.804909 |
| O | -1.850267 | 0.730531 | -0.670046 |
| O | -5.198088 | -0.399295 | -0.366669 |
| O | 2.217748 | 0.195544 | -3.181188 |
| O | 1.181866 | 1.079556 | -1.383862 |
| O | 0.479597 | 1.945029 | 1.098284 |
| O | 3.025966 | 0.823683 | 1.804935 |
| O | 1.850265 | -0.730551 | 0.670022 |
| O | 5.198057 | 0.399405 | 0.366734 |
| C | -2.218630 | -0.700710 | 2.056122 |
| C | -3.579857 | -0.964390 | 1.368638 |
| C | -3.950346 | 0.003070 | 0.217127 |
| C | -2.851603 | -0.013427 | -0.860401 |
| C | 2.218648 | 0.700657 | -2.056123 |
| C | 3.579857 | 0.964397 | -1.368632 |
| C | 3.950352 | -0.003028 | -0.217094 |
| C | 2.851569 | 0.013447 | 0.860395 |
| H | -3.573215 | -1.974587 | 0.944429 |
| H | -4.374258 | -0.907040 | 2.116746 |
| H | -0.902616 | -2.180075 | -0.252747 |
| H | -1.225796 | -1.650719 | -1.655927 |
| H | -4.064472 | 1.017269 | 0.606591 |
| H | -4.905926 | -0.874060 | -1.165671 |
| H | 3.573177 | 1.974602 | -0.944439 |
| H | 4.374272 | 0.907059 | -2.116726 |
| H | 0.902646 | 2.180002 | 0.252705 |
| H | 1.225834 | 1.650634 | 1.655874 |
| H | 4.064532 | -1.017228 | -0.606539 |
| H | 4.905845 | 0.874185 | 1.165709 |

ZnH_2_PO_4_(H_2_O)_5_^+^

| Element | X | Y | Z |
| --- | --- | --- | --- |
| Zn | -0.116003 | -0.007334 | 0.002506 |
| P | -0.174148 | 3.054196 | 0.443306 |
| O | -0.229075 | 0.268682 | 2.079117 |
| O | 1.966902 | 0.530931 | -0.361185 |
| O | 0.024556 | -0.687431 | -2.078687 |
| O | 0.365815 | -2.079304 | 0.284755 |
| O | -0.632628 | 1.874349 | -0.426060 |
| O | -0.855388 | 4.414500 | -0.057279 |
| O | 1.386445 | 3.236398 | -0.032826 |
| O | -2.265034 | -0.297493 | -0.338137 |
| O | -0.299701 | 2.857644 | 1.929022 |
| H | 0.360437 | -0.097301 | 2.746844 |
| H | -0.291052 | 1.266530 | 2.233987 |
| H | -2.545661 | 0.628163 | -0.406109 |
| H | -2.895624 | -0.747921 | 0.236343 |
| H | 0.105633 | -2.674402 | 0.997469 |
| H | 0.399172 | -2.587433 | -0.536160 |
| H | 0.719325 | -0.222332 | -2.563343 |
| H | -0.789605 | -0.593536 | -2.590140 |
| H | 2.750172 | 0.128564 | 0.030453 |
| H | 2.069976 | 1.504116 | -0.317685 |
| H | 1.823884 | 4.032958 | 0.295369 |
| H | -1.412504 | 4.831943 | 0.612157 |

ZnH_4_(PO_4_)_2_(H_2_O)_4_

| Element | X | Y | Z |
| --- | --- | --- | --- |
| Zn | 0.000000 | 0.000000 | 0.000000 |
| P | 0.152977 | -3.130857 | 0.356032 |
| P | -0.152977 | 3.130857 | -0.356032 |
| O | -0.284540 | -0.455317 | 2.109886 |
| O | 2.189632 | -0.432624 | 0.007450 |
| O | -0.345166 | -1.942996 | -0.451249 |
| O | 1.742130 | -3.234111 | -0.067878 |
| O | -0.430448 | -4.514080 | -0.254821 |
| O | -0.014926 | -3.096664 | 1.851717 |
| O | 0.284540 | 0.455317 | -2.109886 |
| O | -2.189632 | 0.432624 | -0.007450 |
| O | 0.345166 | 1.942996 | 0.451249 |
| O | -1.742130 | 3.234111 | 0.067878 |
| O | 0.430448 | 4.514080 | 0.254821 |
| O | 0.014926 | 3.096664 | -1.851717 |
| H | 2.310194 | -1.385539 | -0.157079 |
| H | 2.531911 | 0.024986 | -0.769373 |
| H | -0.156510 | -1.427231 | 2.270638 |
| H | 0.287098 | 0.042540 | 2.703471 |
| H | 2.210142 | -3.939155 | 0.395945 |
| H | -1.064804 | -4.916647 | 0.349523 |
| H | -2.310194 | 1.385539 | 0.157079 |
| H | -2.531911 | -0.024986 | 0.769373 |
| H | 0.156510 | 1.427231 | -2.270638 |
| H | -0.287098 | -0.042540 | -2.703471 |
| H | -2.210142 | 3.939155 | -0.395945 |
| H | 1.064804 | 4.916647 | -0.349523 |

ZnH_3_(PO_4_)_2_(H_2_O)_4_^−^

| Element | X | Y | Z |
| --- | --- | --- | --- |
| Zn | -0.370832 | 0.380948 | 1.146048 |
| P | 0.423424 | -2.495001 | 1.339117 |
| P | 0.732164 | 2.634847 | -0.941206 |
| O | -0.801187 | 0.007543 | 2.983611 |
| O | 3.131254 | -0.117355 | -0.339980 |
| O | -1.636607 | 0.256223 | -2.624763 |
| O | 0.995624 | -1.122308 | 0.881272 |
| O | 1.627077 | -3.593189 | 1.246186 |
| O | 0.227764 | 2.030970 | 0.375718 |
| O | 0.730744 | 4.255532 | -0.745230 |
| O | 2.304038 | 2.354946 | -1.075985 |
| O | -1.872860 | -0.508890 | -0.019093 |
| O | 0.174178 | -2.403805 | 2.911583 |
| O | -0.762600 | -2.970534 | 0.534391 |
| O | -0.044139 | 2.314869 | -2.195827 |
| H | 3.809836 | -0.076887 | 0.340155 |
| H | 2.374553 | -0.594692 | 0.088033 |
| H | -1.106109 | -0.357616 | -3.140985 |
| H | -1.060486 | 1.063005 | -2.517236 |
| H | -1.667859 | -1.473061 | 0.053389 |
| H | -1.844169 | -0.271952 | -0.978563 |
| H | -1.715695 | 0.166316 | 3.228265 |
| H | -0.243161 | -1.499158 | 3.120373 |
| H | 1.360918 | -4.282074 | 0.628349 |
| H | 2.590279 | 1.440136 | -0.821715 |
| H | 0.261422 | 4.645928 | -1.489436 |

ZnHPO_4_(H_2_O)_5_

| Element | X | Y | Z |
| --- | --- | --- | --- |
| Zn | -0.066067 | -0.146578 | 0.010683 |
| P | 0.404165 | 2.822842 | -0.364518 |
| O | -0.219958 | -0.026087 | 2.216002 |
| O | 2.074684 | 0.222606 | 0.514385 |
| O | 0.317670 | -0.239857 | -1.910062 |
| O | 0.318291 | -2.336363 | -0.241830 |
| O | -0.599576 | 1.849183 | 0.308696 |
| O | 0.416452 | 2.501126 | -1.934678 |
| O | 1.785856 | 2.793620 | 0.254429 |
| O | -0.229928 | 4.303460 | -0.294059 |
| O | -2.281003 | -0.243032 | 0.011167 |
| H | 2.644083 | -0.076804 | -0.203560 |
| H | 2.137635 | 1.233271 | 0.490052 |
| H | -0.365392 | 0.935013 | 2.131807 |
| H | 0.672282 | -0.123238 | 2.572518 |
| H | 0.557816 | -2.061898 | -1.152590 |
| H | 1.066778 | -2.811685 | 0.134009 |
| H | -2.360084 | 0.723211 | 0.091001 |
| H | -2.727604 | -0.630449 | 0.772076 |
| H | -0.455789 | -0.379640 | -2.466174 |
| H | 0.473159 | 1.518960 | -2.099329 |
| H | 0.456112 | 4.967210 | -0.160213 |

Zn_2_H_2_(PO_4_)_2_(H_2_O)_4_

| Element | X | Y | Z |
| --- | --- | --- | --- |
| Zn | 0.456152 | 0.220927 | -0.305138 |
| Zn | 0.892024 | -2.546258 | -3.150175 |
| P | 2.893905 | -0.763130 | -1.709835 |
| P | -1.452836 | -1.857966 | -1.443957 |
| O | 0.865338 | -1.259011 | 1.041218 |
| O | 2.113983 | 0.499570 | -1.211017 |
| O | 2.351962 | -1.263606 | -3.079024 |
| O | 2.891667 | -1.864975 | -0.641941 |
| O | 1.528736 | -2.899562 | -5.111485 |
| O | -0.966229 | -2.187857 | -2.895084 |
| O | -1.224315 | -0.360235 | -1.092831 |
| O | -0.806802 | -2.803190 | -0.422180 |
| O | -3.065803 | -1.994185 | -1.449586 |
| O | 1.357780 | -3.853970 | -1.652241 |
| O | 4.396252 | -0.274923 | -2.063385 |
| O | -0.605463 | 1.927068 | 0.276391 |
| H | -1.466366 | 1.719569 | -0.121722 |
| H | -0.350201 | 2.813703 | -0.002721 |
| H | 2.079933 | -3.361341 | -1.183966 |
| H | 0.559569 | -3.742080 | -1.074000 |
| H | 4.873456 | 0.028910 | -1.283321 |
| H | -3.354468 | -2.897931 | -1.618262 |
| H | 1.730603 | -1.583237 | 0.680664 |
| H | 0.205754 | -1.949890 | 0.774023 |
| H | 2.251734 | -2.253277 | -5.159838 |
| H | 0.952877 | -2.764978 | -5.872709 |

**References**

[1] Black JR, Kavner A, Schauble EA (2011) Calculation of equilibrium stable isotope partition function ratios for aqueous zinc complexes and metallic zinc. Geochimimica et Cosmochimica Acta 75: 769-783.
